# Development of a Novel Multi-Epitope Vaccine Against Crimean-Congo Hemorrhagic Fever Virus: An Integrated Reverse Vaccinology, Vaccine Informatics and Biophysics Approach

**DOI:** 10.3389/fimmu.2021.669812

**Published:** 2021-06-16

**Authors:** Muhammad Tahir Ul Qamar, Saba Ismail, Sajjad Ahmad, Muhammad Usman Mirza, Sumra Wajid Abbasi, Usman Ali Ashfaq, Ling-Ling Chen

**Affiliations:** ^1^ College of Life Science and Technology, Guangxi University, Nanning, China; ^2^ NUMS Department of Biological Sciences, National University of Medical Sciences, Rawalpindi, Pakistan; ^3^ Department of Microbiology and Pharmacy, Abasyn University, Peshawar, Pakistan; ^4^ Department of Chemistry and Biochemistry, University of Windsor, Windsor, ON, Canada; ^5^ Department of Bioinformatics and Biotechnology, Government College University Faisalabad, Faisalabad, Pakistan

**Keywords:** Crimean-Congo hemorrhagic fever, Crimean-Congo hemorrhagic fever virus, vaccine, immunoinformatics, molecular dynamics simulation

## Abstract

Crimean-Congo hemorrhagic fever (CCHF) is a highly severe and virulent viral disease of zoonotic origin, caused by a tick-born CCHF virus (CCHFV). The virus is endemic in many countries and has a mortality rate between 10% and 40%. As there is no licensed vaccine or therapeutic options available to treat CCHF, the present study was designed to focus on application of modern computational approaches to propose a multi-epitope vaccine (MEV) expressing antigenic determinants prioritized from the CCHFV genome. Integrated computational analyses revealed the presence of 9 immunodominant epitopes from Nucleoprotein (N), RNA dependent RNA polymerase (RdRp), Glycoprotein N (Gn/G2), and Glycoprotein C (Gc/G1). Together these epitopes were observed to cover 99.74% of the world populations. The epitopes demonstrated excellent binding affinity for the B- and T-cell reference set of alleles, the high antigenic potential, non-allergenic nature, excellent solubility, zero percent toxicity and interferon-gamma induction potential. The epitopes were engineered into an MEV through suitable linkers and adjuvating with an appropriate adjuvant molecule. The recombinant vaccine sequence revealed all favorable physicochemical properties allowing the ease of experimental analysis *in vivo* and *in vitro*. The vaccine 3D structure was established *ab initio*. Furthermore, the vaccine displayed excellent binding affinity for critical innate immune receptors: TLR2 (−14.33 kcal/mol) and TLR3 (−6.95 kcal/mol). Vaccine binding with these receptors was dynamically analyzed in terms of complex stability and interaction energetics. Finally, we speculate the vaccine sequence reported here has excellent potential to evoke protective and specific immune responses subject to evaluation of downstream experimental analysis.

## Introduction

Hemorrhagic fevers are caused by several distinct families of viruses and referred as viral hemorrhagic fevers (VHFs) (1). Hemorrhagic fever associated viruses usually found in moderate and tropic environments and can affect individuals of both sexes and all ages (2). Among these viruses, Crimean-Congo hemorrhagic fever virus (CCHFV), which is ssRNA tick-borne infectious virus, has the potential of causing serious outbreaks of hemorrhagic fever in humans (3). The CCHFV disease is prevalent in more than 30 countries, majorly across the Middle East, Eastern Europe, Asia, and Africa (4). In China, the Xinjiang strain of CCHFV is well known for causing local Xinjiang fever (5).

CCHFV is a *Nairovirus* from the family of *Bunyaviridae*, which transmit to humans through domestic livestock and wild organisms (6). Human beings can catch the virus by the bite of infected animals and exposure to contaminated tissues or blood (7, 8). CCHFV is a negative-sense single-stranded RNA virus and its genome comprised of three particular fragments indicated as: S, M, and L, for small (nucleocapsid protein), medium (glycoproteins), and large (RNA-directed RNA polymerase enzyme), respectively (4, 8). The structural glycoproteins make spikes on the virus surface and facilitate entry into the host cell. Like other *Noroviruses*, CCHFV also encodes a glycoprotein-38 (GP38). The GP38 protein is key in interactions with the vertebrates/tick hosts and critical in cell tropism. It also has an additional key role in CCHFV associated immune response (9, 10). Crystal structure analysis of the glycoprotein demonstrates a unique fold majorly composed of a tri-helical bundle and a β-sandwich. The molecular weight of GP-38 is 38 kDa, and it shares no structural or sequence similarity with the rest of cellular and viral proteins (9, 11). The RNA-directed RNA polymerase enzyme functions in replication and ascription of the viral genome, whereas the nucleocapsid protein is imported in establishing the infection (12).

Major symptoms of CCHFV infection usually involve headache, diarrhea, high fever, myalgia, ecchymosis, epistaxis, emesis, and bleeding gums (4, 8). Ribavirin is usually employed as therapeutic medication; however, the drug use is controversial especially in the later phases of the virus infectious cycle (13, 14). Besides, several vaccine studies have been done in the recent past but due to high toxicity, less protection in case of strain sequence variability, and safety concerns, till, to date, no licensed vaccines are available to fight CCHFV infection (15).

The use of computational immunology and vaccine informatics approaches to engineer a multi-epitope vaccines which are free from allergic, toxic, and unwanted peptide fragments are gaining popularity and now routinely used before experimental vaccinology (16–18). These approaches are successfully applied for number of bacterial, viral, and infectious pathogens (19–26). The main objective of immunoinformatics is to underline immunodominant, safe, and antigenic epitopes, which can evoke strong and safe immunological responses against the pathogen and fulfil all vaccine candidacy benchmarks (27). As the CCHFV is associated with significantly elevated mortality and morbidity across the globe, computational studies needed to develop a hypothetical vaccine construct that can easily be tested for protection against CCHFV in further experiments (28).

Recently, Khan et al. proposed a multi-peptide vaccine candidate using computational approached against CCHFV by targeting only its glycoprotein (28). Since the vaccine constructs have high rate of failure in subsequent analyses and CCHFV exhibits extensive glycoproteins sequence diversity across strains, therefore, more comprehensive vaccine constructs need to be developed from multiple/conserved proteins of CCHFV. Thus, the present study is performed in order to underline the epitopes of CCHFV and highlights the antigenic determinants of N, RdRp, Gn/G2, and Gc/G1 proteins, which can be substantial target for the synthesis of a comprehensive construct to combat CCHFV associated infections. Different bioinformatics tools were employed to predict and evaluate the antigenic/conserved determinants from the multiple proteins of CCHFV, which were integrated further with β-defensin to initiate and augment a life-long immunogenic potential. In order to understand the binding pattern of the construct to innate immune receptors TLR2 (29) and TLR3, blind docking of the construct with these receptors was performed. Further to assess the structural stability and dynamics of the construct, the complexes were subjected to molecular dynamic simulations. Lastly, the binding energies of complexes and all noticeable amino acid residues critical for maintaining complex stability were evaluated in order to validate intermolecular forces of interactions. Conclusively, the current study provides exceptional and novel outcomes for the experimentalists to develop an effective vaccine to combat and control CCHFV infection.

## Materials and Methods

**Figure 1:**
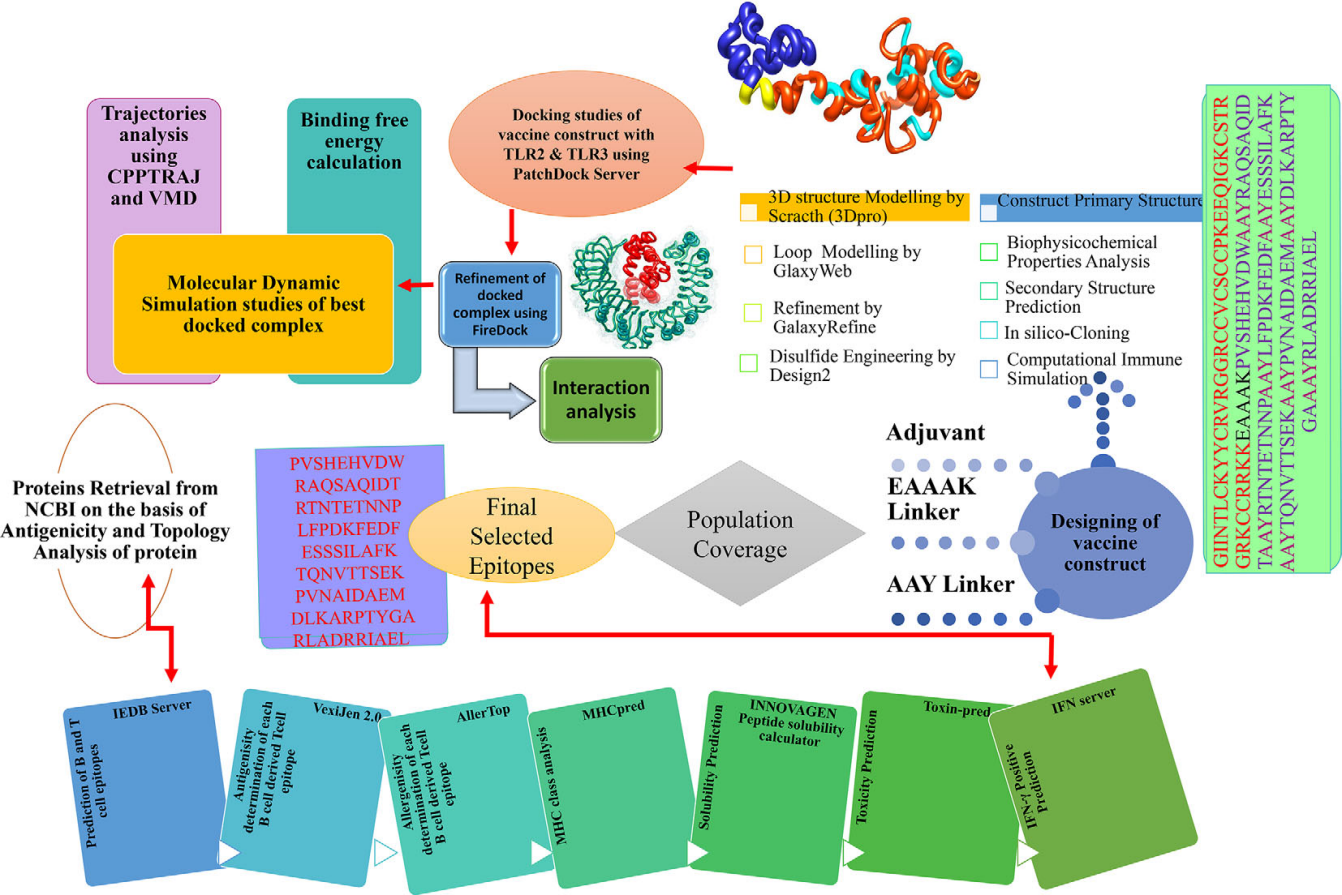
*In silico* approaches implemented to design vaccine construct against CCHFV. Multi-Epitopes Vaccine Design.

The comprehensive *in silico* analysis performed in this study to design a multi-epitope vaccine (MEV) based on the multiple CCHFV proteins is presented in **Figure 1**.

### B-Cell and T-Cell Epitopes Prediction

For epitope screening, the primary amino acid sequence of N, RdRp, Gn/G2, and Gc/G1 proteins were retrieved from complete proteome (Taxonomy ID: 980519) of CCHFV from NCBI (30). The prediction of both B-cell and T-cell epitopes was done *via* Immune Epitope Data Base (IEDB) server (31). Linear B cell epitopes were predicted using Bepipred Linear Epitope Prediction 2.0 method (32), and those with a prediction score of >0.5 were considered in T-cell epitopes mapping. Shortlisting of T-cell epitopes was made based on their strong association with a reference set of major histocompatibility complex (MHC): MHC- I and MHC- II alleles. Epitopes of minimum percentile score are high-affinity binders of the MHC alleles. Filtered epitopes were further used in MHCPred 2.0 (33) for the interpretation of their potential binding compatibility to the highly prevalent HLA II DRB*0101 allele in human populations and those having IC50 value < 100 nM were graded as efficient binders of DRB*0101 (34). Virulent epitopes were predicted through VirulentPred (35) using a cutoff value of 0.5. Further, antigenic epitopes were forecasted using VaxiJen 2.0 (36). AllerTop 2.0 (37) was used to disclose allergenic epitopes and toxic peptides were discarded *via* ToxinPred (38). To evaluate tendency of inducing IFN-γ responses, the filtered non-toxic epitopes were examined by IFN epitope server (39), and only IFN-γ positive epitopes were opted for further analysis. Finally, IFN-γ–inducing epitopes were investigated *via* IEDB population coverage analysis (40).

### Multi-Epitopes Vaccine Design

AAY linkers were utilized to associate all processed epitopes to produce a MEV construct (18). To design a complete vaccine ensemble, the processed sequence was coupled with immunogenic β-defensin in order to maximize immunogenicity of MEV (41). The physicochemical properties of the complete vaccine unit were estimated using the ProtParam tool of EXPASY server (42). The MEV construct was evaluated for antigenicity, immunogenicity, solubility, and allergenicity using VaxiJen v2.0/ANTIGENpro (36, 43), IEDB/ProPred (31, 44), Proetin-sol/SOLpro (45, 46), and AllerTOP/Allergen FP (37, 47), tools, respectively. SOPMA (48) was used to analyze secondary structure of MEV. As the MEV is constructed by joining different epitopes from different proteins and no suitable template found during homology search, an *ab initio* modeling of the vaccine was implemented to build its 3D structure with the help of 3Dpro of SCRATCH protein server (43). Later on, loop modeling in the 3D structure of vaccine was carried out by using Galaxy Loop (49), and then refined through Galaxy Refine (50) of Galaxy Web server. Disulfide bonds were introduced in the vaccine 3D structure to achieve structural stability and were done by disulfide by Design 2.0 (51). The MEV protein sequence was reverse translated into gene sequence for *in silico* expression cloning analysis. First, the codon usage of the vaccine was adjusted as per *E. coli* K12 expression system utilizing (JCat) Java Codon Adaptation Tool (52), and expression was determined by (CAI) Codon adaptation index value and GC percentage. The RNA secondary structure of an optimized sequence was predicted using Mfold server (53). The cDNA of the vaccine was then cloned into pET-28a (+) expression vector using SnapGene (https://www.snapgene.com/).

### Host Immune System Simulation

The MEV was subjected to C-ImmSim server (54) to computationally elucidate its potential to trigger host immune system. This server operates by machine learning and position-specific scoring matrix (PSSM) to evaluate host immune responses toward antigen (54). Three anatomical parts are associated with the host immune system, including thymus, bone marrow, and lymph nodes. The input criterion for the immune simulations included a standard volume of 10, random seed 12345, 100 steps, number of injections calibrated to three (4 weeks space in each dose), and HLA (A0101, A0101, B0702, B0702, DRB1_0101, DRB1_0101) and keeping other features as default.

### Molecular Docking

At this phase, molecular docking of designed MEV with human TLR2 and TLR3 (PDB ID: 5D3I and 1ZIW) was performed to assess the vaccine’s affinity to the said receptors. TLR3 is a pattern recognition receptor family transmembrane protein. RNA viruses’ infection is detected and responded by eliciting the expression of NF-kB (Nuclear Factor kappa-light-chain-enhancer) and interferon regulatory transcription factor (IRF3). In contrast to other TLRs, TLR3 recruits TIR-domain-adapter-inducing interferon-β (TRIF). The innate immune system is stimulated and activated due to IRF3, which increases type I interferons development, that substantially induces adaptive immunity (55, 56). Vaccine was blindly docked to the TLR2 and TLR3 receptors through PATCHDOCK web server (57). Docked solutions resulting from docking were instantly refined for interactions using Fire Dock server (58). This imparts effective refinement of the substructure of PATCHDOCK complexes. Complexes with low global energies were selected and visualized in UCSF Chimera (59) for in-depth visualization.

### Molecular Dynamics Simulation

To better understand dynamics, stability, and structural integrity of docked complexes, molecular dynamics simulation was run over a period of 100 ns. This analysis was distributed into three phases, including complexes parameterization, pre-processing, and simulation production. During the initial stage, an antechamber module of AMBER18 (60) was utilized to generate parameters for vaccine and receptors. Complexes were solvated in 12 Å TIP3P solvation box and accomplished through Leap module of AMBER. Ff14SB force field (61) was used to define both receptors and vaccine parameters. To neutralize charge density, Na+ ions were introduced to systems as counter ions. During the pre-processing step, several rounds of complexes energy minimization were done; hydrogen atoms energy minimization for 500 steps, water molecules energy minimization for 1000 steps with restrain of 200 kcal/mol—Å^2^ on the remaining system, 1000 steps of energy minimization of all atoms exception to 5 kcal/mol—Å^2^ restraint on alpha carbon atoms, and 300 steps energy minimization on non-heavy atoms with the restraint of 100 kcal/mol—Å^2^ on rest of the complex. Systems were later heated to 300K (NVT ensemble). Temperature on the systems was maintained through Langevin dynamics (62), and hydrogen bonds were restricted using SHAKE algorithm (63). The complexes were equilibrated for 1000 ps. The system was compressed with NPT ensemble constraining Cα atoms of 5 kcal/mol energy–Å^2^. The production run for each system was accomplished for 100 ns. CPPTRAJ module (64) of AMBER was used for simulation trajectories analysis. The visualization and analysis of MD simulation trajectories were done in VMD software (65).

### Radial Distribution Function

The radial distribution function describes probability distribution to determine the center of a particle in the distance “r” from the center of a reference (66). This parameter provides packing structures information and detail long-range inter-particle correlation and how they are organized. Radial distribution function plots of the interactions involved in giving specific patterning of the vaccine-TLR systems were extracted using AMBER’s PTRAJ module. The idea was to look for the stability of each system’s interactions during the simulation and to predict whether interacting pattern remains constant over a period of time.

### Computing Binding Free Energies

The binding energies and solvation free energies for vaccine, receptors, and complexes were estimated by using MMPBSA.py module (67) of AMBER18. The mean value of these energies was evaluated as the overall binding free energy of the systems. Mathematical interpretation of MMPBSA binding energy can be done as:

$$\Delta G_{bind,\;solv}=\Delta G_{bind,\;vaccum}+\Delta G_{solv,\;receptors}-(\Delta G_{solv,\;vaccine}+\Delta G_{solv,\;receptors})$$

Estimation of binding energy for all three components was made either by Generalized Born (MMGBSA) or Poisson Boltzman (MMPBSA) equation. The solvation energy is further split as:

$$\Delta G_{solv}=G_{electrostatic,\;\epsilon =80}-G_{electrostatic,\;\epsilon =1}+\;\Delta G_{hydrophobic}$$

whereas the vacuum phase energy can be described by the following equation,

$$\Delta G_{vacuum}=\Delta E_{molecular\;mechanics}-T(\Delta S)$$

## Results

### Prediction of Potential Epitopes

**Figure 2:**
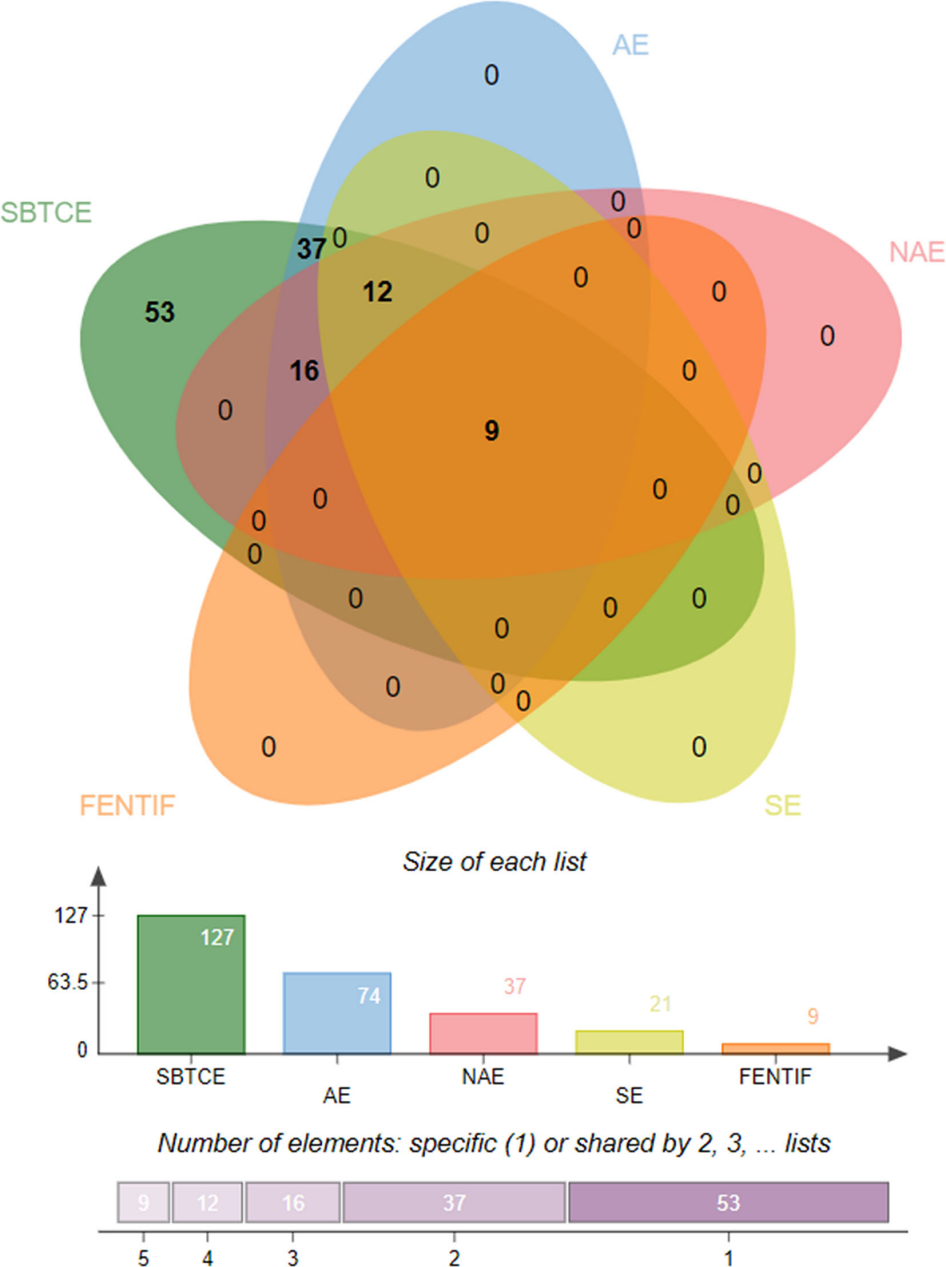
Venn diagram presenting the number of epitopes filtered at different phases. SBTCE, Shared B and T cell epitopes; AE, Antigenic Epitopes; NAE, Non-Allergen Epitopes; SE, Soluble Epitopes; FENTIF, Final set of Epitopes Nontoxic and IFN gamma positive producer.

Prediction of potential antigenic epitopes was done based on the amino acid sequences of N, RdRp, Gn/G2, and Gc/G1 proteins of CCHFV and then engineered into a MEV. The predicted epitopes were prioritized using several steps, each ensured selection of potential epitopes that fulfil requirements of an effective MEV candidate. An ideal multi-epitope vaccine should contain B and T-cell epitopes to stimulate an extensive immune response network. Initially, the proteins were analyzed for B-cell epitopes, and total 10 epitopes were predicted from N, 6 from RdRp, 5 from Gn/G2, and 11 from Gc/G1 proteins. The predicted epitopes were 5 to 100 amino acids long. The B-cell epitopes then subsequently investigated for T-cell epitopes and only strong binders to both MHC-I and MHC-II were selected based on the lowest percentile score. The selected epitopes were then examined for common peptides. For selection of T-cell epitopes, only the reference set of MHC alleles was opted. Each epitope further go-through sequence similarity check with human proteome to discard epitopes homologous with the host. This is a pre-requisite, as any epitope similar to the host may lead to strong autoimmune reactions and instead of protection, the MEV may deteriorate the host health. To facilitate experimental testing of MEV before clinical trials, the epitopes sequence identity was also tested against the mouse proteome and results revealed no similarity. All the filtered epitopes were also found free from transmembrane helices, that might ease the cloning and expression analysis. One key factor which is essential for downward analysis is the epitopes’ binding ability to the host immune system products, which means their antigenicity potential. All the filtered epitopes were predicted to be strongly antigenic. Moving forward, only epitopes were selected that have a high capacity of adhesion. The epitopes were also found non-allergic, thus reducing the chances of causing vaccine-associated allergic responses. Finally, careful evaluation resulted into 31 epitopes which were safe and non-allergic (**Table 1**). Next, the epitopes were subsequently prioritized based on their solubility (21 in number), non-toxicity (18 in number) and IFN-gamma producing ability (9 in number) (**Figure 2**). Importantly, the overall population coverage of these epitopes was 99.74% (**Table S1**).

**Table 1:** Number of epitopes obtained at each step of epitope mapping phases.

Proteins	B-cell	MHC I	P.R	MHC II	P.R	Common Peptide	Antigenicity	Allergenicity	MHC- Pred	Solubility	Toxicity	IFN-gamma
**N**	LILNRGGDENPRG PVSHEHVDWCRE	GPVSHEHVDW	0.09	PRGPVSHEHVDWCRE	52	PVSHEHVDW	1.7725	Non-Allergen	23.28	Soluble	Non-Toxic	Positive
	KNSSALRAQSAQIDT	KNSSALRAQS	9.5	KNSSALRAQSAQIDT	0.77	KNSSALRAQS	0.5185	Non-Allergen	58.34	Soluble	Non-Toxic	Positive
	NLRTNTETNNP	RTNTETNNP	3.3	NLRTNTETNNP	19	RTNTETNNP	0.0128	Non-Allergen	13.12	Soluble	Non-Toxic	Positive
**RdRp**	LFPDKFEDFLDRTQLH PEFRDLTPDFSLTQKVHFKRNQIPSV	LFPDKFEDF	0.06	LFPDKFEDFLDRTQL	7.1	LFPDKFEDF	0.4024	Non-Allergen	55.21	Soluble	Non-Toxic	Positive
	ATLPESVEAVPVIERKMFPLPETPLSEVH SIERIMENFTRLMHEGRPSTKGKDKEPAE QDNHQNAIEHESSSILAFKDYGERGIVEE NHMRLSEEDQLET	ESSSILAFK	0.01	ESSSILAFKDYGER	4.3	ESSSILAFK	0.8525	Non-Allergen	32.96	Soluble	Non-Toxic	Positive
	ENLDRITDEFERTKFKHELTQNVTTSEK	LTQNVTTSEK	0.3	KFKHELTQNVTTSEK	9.3	TQNVTTSEK	0.578	Non-Allergen	48.98	Soluble	Non-Toxic	Positive
**Gn/G2**	PVNAIDAEMHDLNCSYN	PVNAIDAEM	6.9	PVNAIDAEMHDLN	6.1	PVNAIDAEM	0.6089	Non-Allergen	9.73	Soluble	Non-Toxic	Positive
**Gc/G1**	HFHSKRVTAHGDTPQLDLKARPTYGA	DLKARPTYGA	2.8	DTPQLDLKARPTYGA	25	DLKARPTYG	1.5891	Non-Allergen	53.95	Soluble	Non-Toxic	Positive
	RGLFKYRHLKDDEETGYRRIIEKL NNKKGKNKLLDGERLADRRIAELFSTKT	RLADRRIAEL	0.1	RLADRRIAELFSTKT	7.4	LADRRIAEL	0.529	Non-Allergen	10.26	Soluble	Non-Toxic	Positive

*P.R representing percentile rank.

### MEV Designing

**Figure 3:**
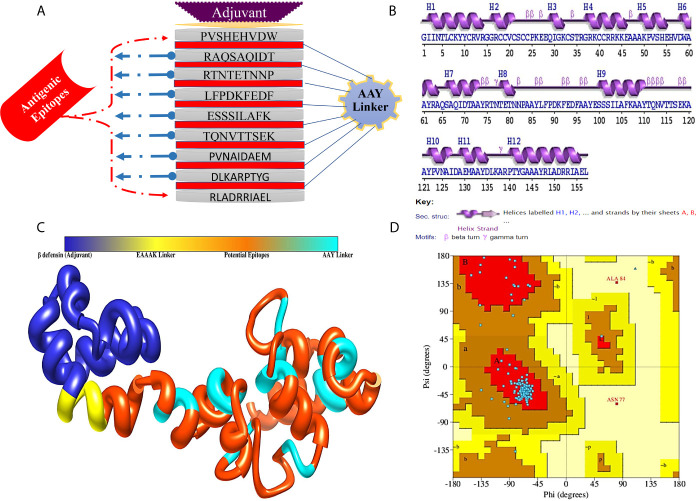
**(A)** Schematic representation of MEV construct for CCHFV, **(B)** Secondary structure of MEV construct, **(C)** Predicted 3D structure of designed MEV construct and **(D)** Ramachandran plot of MEV construct.

The MEV construct was designed by combining filtered epitopes from all the target proteins. Designing of such vaccines is a promising technique, as it circumvents many limitations associated with the whole organism-based vaccines, as well as sub-unit vaccines. Moreover, due to limited antigenic capabilities of induvial epitope-based peptide vaccines, MEV designed from multiple conserved proteins strengthen immune stimulation and generate strong and specific immune response. To combine the selected epitopes, AAY linkers were employed. These linkers increased α-regions and reduced β-, turn-, and coil-regions, thus making the epitopes lesser flexible. To the N-terminus, EAAAK linker is rigid and keep the functional domain separated and allow its proper presentation to the host immune system. β-defensin was attached as adjuvant, which is a powerful immune adjunct as it significantly amplifies lymphokines secretion leading to the regulation of T cell-mediated immune response and the synthesis of antigen-specific immunoglobulin (68). MEV is schematically illustrated in **Figure 3A**. The sequence of designed MEV sequence is:

“GIINTLCKYYCRVRGGRCCVCSCCPKEEQIGKCSTRGRKCCRRKKEAAAKPVSHEHVDWAAYRAQSAQIDTAAYRTNTETNNPAAYLFPDKFEDFAAYESSSILAFKAAYTQNVTTSEKAAYPVNAIDAEMAAYDLKARPTYGAAAYRLADRRIAEL”.

### Physicochemical Properties of MEV

The MEV is 157 amino acids long and has molecular weight of 17.44 7 kDa. Generally, vaccine of molecular weight < 110 kDa, is supposed to be feasible during purification process. Theoretical pI scores the vaccine was 8.90. This can aid in tracking the vaccine on a 2D gel. The aliphatic index score was 61.21, indicating its thermostable nature of the vaccine. The vaccine has 17 negatively charged, and 24 positively charges residues. Later, GRAVY score was predicted for the vaccine, and that was −0.477, indicating hydrophilicity. The half-life of the vaccine in reticulocytes of mammals, *in vitro* was 30 h, and *Escherichia coli in vivo >*10* h*, and yeast *in vivo* > 20 h. The net entropy observed was 17.01 and have no transmembrane helices (alpha-helical transmembrane protein, 0.04 and beta-barrel transmembrane, 0.006); hence, no difficulties can be anticipated in cloning and expression analysis. Next, the MEV construct was evaluated for antigenicity, immunogenicity, solubility, and allergenicity by using multiple tools to ensure the accuracy. The results revealed that designed MEV is highly antigenic (scores: 0.50 and 0.87), immunogenic, soluble (scores: 0.59 and 0.98), and non-allergic.

### Modeling and Refinement of MEV

The secondary structure analyses revealed that 50.32% (69) residues form α-helix, while 8.28% (13), 4.46% (7), and 36.94% (58) establish β-strand, β-turn, and random coils, respectively (**Figure 3B**). Since the MEV is combination of epitopes derived from different proteins of CCHFV, there was no appropriate template for homology modeling of the MEV was available, thus the 3D structure of the MEV was modeled *ab initio* using 3Dpro predictor tool. The 3D structure of MEV is demonstrated in **Figure 3C**. The MEV 3D acquired 90.3% of residues in the Ramachandran plot favored, 8.3% in additionally allowed regions, 0.0% generously allowed regions, and 1.4% residues belonged to the disallowed regions (**Figure 3D**). As there were loops in the structure, several rounds of proper loop modeling were performed on the structure. Five loops of amino acid residues were subjected to loop modeling: Tyr10-Glu28, Cys33-Gly37, Alas48-Val57, Thr76-Glu99, Thr116-Ala121, and Leu136-Tyr139. Afterward, loop modeled structure underwent refinement for structural contractions and relaxations to get a processed structural model. Among the generated models, the first model was chosen for subsequent analyses (**Table S2**). Model selection was done based on a better Ramachandran favored score, improved clash score of 1.9, lowest stable galaxy energy of −3151.35, and refined values of MolProbity (1.266). Similarly, poor rotamers were missing in the structure as compared to its original conformation. Ramachandran’s statistics for the refined 3D structure were observed in following order: 0% in generously allowed regions, 90.3% in Ramachandran favored residues, 0% in disallowed regions, and 9.4% in additionally allowed regions.

### Disulfide Engineering and *In Silico* Cloning

**Figure 4:**
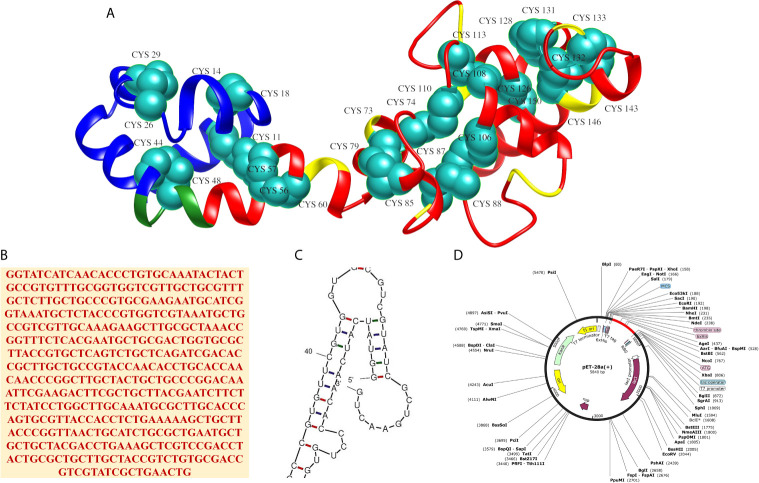
**(A)** 3D structure of the MEV after disulfide engineering with mutated residue are shown in cyan-green spheres, **(B)** Reverse translated primary DNA sequence of the MEV, **(C)** Closeup view of start-site of MEV RNA structure (full structure is given in **Figure S1**) and **(D)** *In silico* cloning of MEV (shown in red) in pET28a(+) vector.

Disulfide engineering of the MEV was accomplished for 14 pairs of amino acid residues. These pairs were: Cys11-His56 (χ3 angle,+126.49, energy value, 7.71 kcal/mol), Arg14-Cys18 (χ3 angle, −71.10, energy value, 6.37 kcal/mol), Lys26-Gln29 (χ3 angle, −100.70, energy value, 2.68 kcal/mol), Lys44-Ala48 (χ3 angle,+88.42, energy value,5.95 kcal/mol), Val57-Ala60 (χ3 angle, −98.14, energy value, 2.22 kcal/mol), Ala73-Glu79 (χ3 angle, −83.45, energy value, 2.75 kcal/mol), Tyr74-Tyr110 (χ3 angle, −104.92, energy value, 2.48 kcal/mol), Ala85-Phe88 (χ3 angle,+125.58, energy value,4.80 kcal/mol), Leu87-Phe106 (χ3 angle, −113.76, energy value, 3.52 kcal/mol), Ala108-Asn113 (χ3 angle, +118.50, energy value, 4.11 kcal/mol), Ala126-Ala150 (χ3 angle, +91.62, energy value, 4.67 kcal/mol), Asp128-Met131 (χ3 angle, +113.53, energy value, 1.34 kcal/mol), Ala132-Ala146 (χ3 angle, −58.52, energy value, 7.37 kcal/mol), and Ala133-Gly143 (χ3 angle, +118.04, energy value, 4.81 kcal/mol). As these residues had highly unstable energy level of >1.34 kcal/mol and χ3 angle out of range (< −87 and > + 97). The mutated MEV 3D structure is shown in **Figure 4A**. Further, *in silico* cloning of MEV was achieved in pET28a(+) expression vector to assist genetic engineers and molecular biologist in cloning the MEV experimentally and get a high possible expression. Reverse translation and codon optimization of the MEV was done as per *E. coli* K12 system to optimize the expression (**Figure 4B**). The CAI value of the MEV was 1, indicating ideal expression. The GC content was 54.56% almost equal to the *E. coli* K12. In addition, no hairpin loop or pseudoknot was found at the starting site of MEV RNA secondary structure (**Figure 4C**). The cloned construct is illustrated in **Figure 4D**.

### MEV Immune Simulation

**Figure 5:**
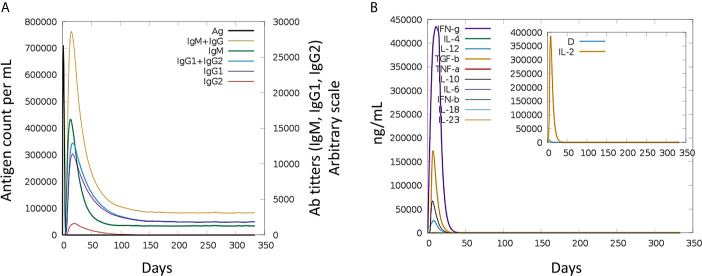
Host immune system simulation in response to. The immune response (generation of Igs) by antigen exposure are shown in **(A)**, and cytokines and interleukins production in different stated with Simpson index is shown in **(B)**.

Immune simulation of the host immune system in response to the MEV revealed robust primary and secondary immune reactions. The host immune system response in terms of different antibodies titers and cytokines and interleuckins responses to the MEV are illustrated in **Figure 5**. As can be noticed, elevated IgM and IgG + IgG titers to the MEV was witnessed at the primary feedback, subsequently IgG1 + IgG2, IgM, and IgG1 were observed at both primary and secondary levels with prompt antigen clearnace. Moreover, vigorous reactions of high levels of IFN-γ, IL-10, and IL-2 were also observed. These responses demonstrated effective immune system simulation efficiency of the MEV. Furthermore, higher populations of B-cell production and different isotypes, together with memory cells suggests a life-long memory cell synthesis and immunoglobulin class switching. Moreover, helper T-cells plus cytotoxic T-cells and their corresponding memory generation are in close association with the robust response toward the antigen.

### Binding Mode and Interactions of MEV With Immune Receptors

**Figure 6:**
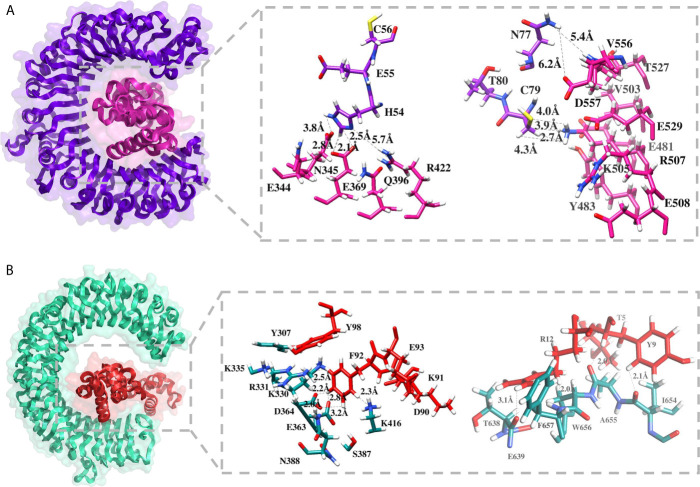
Molecular docking between MEV and TLR receptors. **(A)** Docked conformation and residues interaction map of MEV (shown in pink) to TLR2 (shown in purple), and **(B)** Docked conformation and residues interaction map of MEV (shown in red) to TLR3 (shown in cyan green).

Molecular docking of the designed MEV was carried out with specific immune response receptors, TLR2 and TLR3. Stable interactions with these receptors are essential to produce an effective immune reactions. TLR3 has a marked effect in recognition of virus linked molecular patterns leading to activation of NF-kappa B and type I interferons. For both receptors, 20 docked solutions were generated and ranked based on binding energy (**Table S3**). The assessments were also made on the basis of binding conformations of molecules, with respect to each other through size, area, rigid transformation, desolvation energy, and scoring functions. Subsequently, the complexes were processed to FireDock web server (70) analysis for structural refinement. This was significant in removing false-positive predictions made by initial docking run. Solution 5 of TLR2 (net global energy, −14.33 kJ/mol) and 3 of TLR3 (net global energy, −6.95 kJ/mol) were selected for further analysis. The selected TLR2 solution net energy is the attribute of −42.75 kJ/mol attractive van der Waals energy, and 32.11 kJ/mol of repulsive van der Waals energy, 7.58 kJ/mol of atomic contact energy (ACE), and −5.46 kJ/mol of hydrogen bond energy. In case of TLR3 selected solution, the energy can be split as, −7.70 kJ/mol attractive van der Waals energy, and 1.50 kJ/mol of repulsive van der Waals energy, −2.47 kJ/mol of ACE, and 0.00 kJ/mol of hydrogen bond energy. The configuration of docked MEV and intermolecular interacting residues of the MEV with TLR receptors is illustrated below in **Figure 6**. Visual examination of both solutions indicated strong binding of the MEV at the central cavity of TLR2 preferentially by weak van dar Waals forces and hydrogen bonds. The MEV was detected to form associations with the residues including Ser33, Lys55, Ser56, Val80, Gln79, Gln152, Asn177, Arg316, Glu344, Asn345, Glu369, Arg422, Glu481, Tyr483, Val503, Lys505, Arg507, Thr527, Glu529, Val556, Asp557 of TLR2, and Tyr307, Lys330, Arg331, Lys335, Glu363, Asp364, Ser387, Lys416, Leu440, Tyr465, Lys613, Thr638, Glu639, Ile654, Al655, Trp656, Phe657 of TLR3.

### Molecular Dynamics Simulations

**Figure 7:**
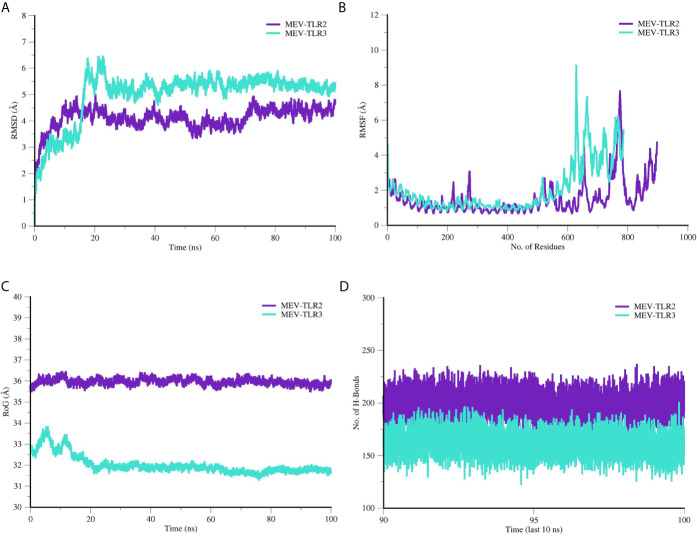
Molecular dynamics simulation analysis of MEV and TLR receptors. **(A)** RMSD, **(B)** RMSF, **(C)** RoG and **(D)** Hydrogen bond analysis.

Molecular dynamics (MD) simulation was further performed on the docked MEV and TLRs complexes to investigate stability and affinity of the systems at thermobaric condition and time (**Figure 7**). MEV and TLRs receptor conformational behavior were evaluated by estimating backbone root-mean-square deviation (RMSD) using the initial structure as a reference. Both the systems were seen in good stability, especially after 20 ns. The mean RMSD of MEV-TRL2 is 4.09 Å (± 0.418), whereas the mean RMSD reported for the MEV-TLR3 is 4.99 Å (± 0.93). Further, from residues flexibility perspective, TLRs were found stable. However, due to the presence of unmodelled loops, the residues displaced limited flexibility. The mean root-mean-square fluctuation (RMSF) of MEV-TLR2 was 1.52 Å (± 0.97) and MEV-TLR3 was 2.02 Å (± 1.39). The folding and compactness of the systems were elucidated through a radius of gyration (RoG) analysis. Like RMSD, a similar trend in systems stability was observed. For instance, MEV-TLR2 mean RoG was 35.95 Å (± 0.14) and MEV-TLR3 RoG was 32.00 Å (± 0.44). After an initial minor change in folding, which is expected to adopt the dynamics environment, both systems achieved higher structure equilibrium toward the end of simulation time. The systems’ stability was evident due to formation of a large number of hydrogen bonds, and therefore, MEV complex attained a more favorable conformation over the period of 100 ns MD simulation time period.

### Radial Distribution Frequency Analysis

**Figure 8:**
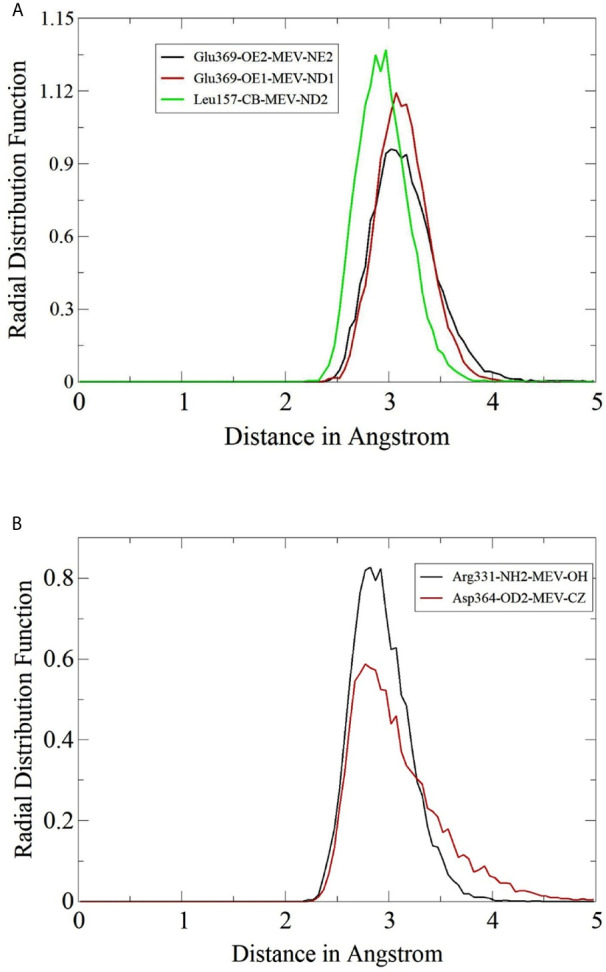
Radial distribution plots for close interactions of MEV and TLRs. **(A)** Plots for MEV and TLR2 and **(B)** Plots for MEV and TLR3.

The hydrogen bonds formation between MEV and TLRs were further studied in term of their radii distribution during simulation time. It was revealed that the binding of MEV to TLR2 is dominated by several interactions leading to strong stability of the complex. RDF plots were generated for some of the close intermolecular contacts and are presented in **Figure 8**. The plot demonstrated the stable interactions of MEV in the pocket of TLRs, and the radii distribution remains uniform throughout the simulation period.

### MMGB/PBSA Binding Free Energies and Residues Decomposition Analysis

The atomic-level interaction energies were investigated using MMGB/PBSA. The MM-PBSA.py module of AMBER18 was used as an end state free energies computation approach as it is computationally easy to perform and more reliable than docking scoring functions (71). Binding free energies for both TLR2 and TLR3 complexes computed *via* MMGBSA and MMPBSA methods are tabulated in **Table 2**. Compared to TLR3, TLR2 complex with MEV was highly stable with total MMGBSA binding free energy (ΔG_total_) of −260.14 kcal/mol and MMPBSA energy of −309.77 kcal/mol. The system energy is dominated by electrostatic energy (−889.10 kcal/mol in MMGB/PBSA) and favorably supported by van der Waals energy (−224.50 kcal/mol). The system solvation energy is 853.46 kcal/mol in MMGBSA (878.61 kcal/mol of polar solvation energy and −25.15 kcal/mol non-polar solvation energy), and MMPBSA 803.83 kcal/mol (815.54 kcal/mol of polar solvation energy and -11.71 kcal/mol of non-polar solvation energy). Further, the net energy of MEV-TLR2 system was decomposed into residues of TLR2 to underline residues that are hotspot in interactions with the MEV. The following are the hotspot residues for the MEV-TLR2 system; Ser33, Ser56, Lys55, Gln79, Gln152, Asn177, Val80, Gly344, Asn345, Arg422, Tyr483, Val503, Lys505, Arg507, Val556, Asp557, and Glu629.

**Table 2:** Binding free energy calculations of MEV-TLRs systems by MM-GB(PB)SA method.

MEV-TLR2 System				
**Energy Component**	**Net Energy (MMGBSA/MMPBSA)**		**Std. Dev (MMGBSA/MMPBSA)**	**Std. Err. of Mean (MMGBSA/MMPBSA)**
**Van der Waals**	−224.50/−224.50		5.21/5.21	0.57/0.57
**Electrostatic**	−889.10/−889.10		26.31/26.31	2.45/2.45
**Polar solvation**	878.61/815.54		23.81/23.11	2.13/2.12
**Non-polar solvation**	−25.15/−11.71		0.64/0.21	0.05/0.01
**Gas phase**	−1113.60/−1113.60		26.05/26.05	2.17/2.17
**Solvation phase**	853.46/803.83		23.97/24.21	2.20/2.31
**Total**	−260.14/−309.77		7.81/8.71	0.81/1.10
**MEV-TLR3 System**				
**Energy Component**	**Net Energy** **(MMGBSA/MMPBSA)**		**Std. Dev (MMGBSA/MMPBSA)**	**Std. Err. of Mean (MMGBSA/MMPBSA)**
**Van der Waals**	−114.20/−114.20		5.51/5.51	0.55/0.52
**Electrostatic**	−987.49/−987.49		27.93/27.93	2.79/2.79
**Polar solvation**	1068.96/1027.65		25.38/23.99	2.53/2.39
**Non-polar solvation**	−17.45/−15.67		0.60/0.27	0.06/0.02
**Gas phase**	−1101.70/−1101.70		28.51/28.51	2.85/2.85
**Solvation phase**	1051.51/1011.97		25.09/23.92	2.50/2.39
**Total**	−50.18/−89.72		6.88/10.02	0.68/1.00

For TLR3, the total binding energy (ΔG_total_) of −50.18 kcal/mol in MMGBPSA and −89.72 kcal/mol in MMPBSA. In MMGBSA, delta energy for MEV-TLR3, TLR3, and MEV is −70725.09 kcal/mol, −57301.02 kcal/mol, and −13373.87 kcal/mol, respectively. High contribution in MMPBSA was observed from MEV, followed by receptor TLR3, and MEV-TLR3 complex. Total electrostatic energy estimated for the system in both MMGBSA and MMPBSA was −987.49 kcal/mol and dominate the overall energy of the system. This energy can be split into, MEV electrostatic contribution (−9500.50 kcal/mol), TLR3 (−45202.56 kcal/mol), and MEV-TLR3 (−55690.56 kcal/mol). Van der Waals energy is also favorable with net system energy is −144.20 kcal/mol (MEV-TLR3, −6432.18 kcal/mol; TLR3, −5259.64 kcal/mol; and MEV, −1058.32 kcal/mol). Total solvation free energy for the system was noticed unfavorable, i.e. MMGBSA (1051.51 kcal/mol) and MMPBSA (1011.97 kcal/mol) mainly because of polar energy (MMGBSA, 1068.96 kcal/mol and MMPBSA, 1027.65 kcal/mol). Non-polar solvation energy, on the other hand, seems favorable in both MMGBSA and MMPBSA is −1101.70 kcal/mol. List of hotspot residues in MMGBSA and MMPBSA is given in **Table S4**. The following residues are hotspot residues for the MEV-TLR3 system; Hie3, Val5, Asp7, Leu135, Asn143, Ala190, Ser207, Asn236, Thr237, Leu24, Met249, Ala266, Gly291, Ser337, Leu343, Asp390, Arg453, Arg454, Ser463, Thr603, Phe609, Ile619, Phe622, Asn627, Glu628, Thr629, Thr634, Lys637, Tyr638, Arg641, Val642, Arg646, Val649, Lys679, Hie683, Cys686, Trp688, Gln694, Arg704, Lys720, Phe721, Glu722, Val743, and Thr744.

## Discussion

Vaccines are the potential factors in controlling infectious diseases and improving public health. The traditional vaccine development approaches are labor intensive, expensive and time consuming. In addition, failure chances are high in later trials (20). Therefore, researchers are now focusing on cutting edge next generation approaches to facilitate the vaccine development process. These approaches mainly include reverse vaccinology, immunoinformatics, vaccine informatics, and subtractive proteomics. These approaches provide variety of databases, servers and tools, and enable researchers to identify pathogen proteins that are most suitable for vaccine designing, followed by the prediction of highly antigenic, non-toxic, non-allergic, and safe candidate epitopes, which can be further employed in vaccine design process and tested directly in experimental analyses. CCHFV is a global health concern with 10% to 40% mortality rate and un-availability of proper therapeutics (28). To date, no approved vaccine is available against CCHFV. Therefore, present study was designed to construct an effective and novel MEV against CCHFV by utilizing integrated computational pipeline based on reverse vaccinology, vaccine informatics and biophysics approaches. MEV design was preferred because it can induce humoral, innate, and cellular immunity response together. In addition, MEV is safer compared to other types of vaccines (72).

Previously, two different vaccines were designed based on two major structural CCHFV proteins (N and G) (28, 73). Although results of these studies were promising, but the methodology they applied was not rigorous enough, and several extensive computational steps were missing. Deyde et al. studied 13 complete genomes of CCHFV and found that N, RdRp, Gn/G2, and Gc/G1 proteins were highly variable (74). Therefore, the framework applied in this work used deeper and more extensive *in silico* steps and used all four major antigenic proteins of the CCHFV for MEV design. This effort is novel and presenting unique immunodominant epitopes from multiple conserved proteins. An ideal MEV should contain B- and T-cell epitopes to stimulate an extensive immune response network. Hence, immunodominant epitopes from target proteins (N, RdRp, Gn/G2, and Gc/G1) were predicted and analyzed rigorously by employing various approaches. The most potent nine epitopes were chosen based on their immunogenic properties (i.e., antigenicity, allergenicity, toxicity, and cytokine production) for further analyses. The selected epitopes showed 99.74% global coverage. This result suggested that the MEV would be effective on majority of the world population round the globe. Next, the MEV sequence was designed by joining selected epitopes through linkers and adjuvant. An adjuvant (β-defensin) was added to the N-terminal of the MEV along with EAAAK linker, and epitopes were fused together through AAY linkers. β-defensin serves as an excellent adjuvant due to its antimicrobial and immunomodulatory properties, and it has been used in several previous studies (24, 25, 75), whereas AAY Linkers were added to help maintain the function of individual epitopes, so that after being transported into the host body, they can work accurately (76).

To ease follow up experimental analyses of the MEV and allow successful setting of *in vitro* and *in vivo* experiments, physicochemical properties of the vaccine were assessed. The designed MEV was found to be highly antigenic, immunogenic, soluble, thermostable, and non-allergenic, demonstrating the epitope vaccine’s ability to elicit robust immune responses without causing allergic reactions. Tertiary and secondary structures provide information about a protein’s function, interactions with other proteins/ligands, and dynamics (77). The desirable characteristics of MEV improved significantly after predicted 3D structure refinement. The Ramachandran plot analysis shows that 90.3% of residues are in a favored region, with 0% residues in the disallowed region, indicating that the model is of good quality. Additionally, MEV was subjected to disulfide engineering to optimize vaccine molecule structure and confer substantial structural stability (78). Disulfide engineering introduces disulfide bridges into the final MEV construct and significantly increases protein’s thermostability and also aid in the examination of genetic components of the vaccine. The serological immune-reactivity test is one of the first steps to validate a candidate vaccine (69). Recombined protein must be expressed in an appropriate host. Reverse transcription, codon optimization, RNA secondary structure analyses followed by *in silico* cloning revealed that our designed MEV will be expressed at a high level in *E. coli* K12 system.

Theoratically, since the MEV designed by joining multiple B derived T cell epitopes, it should elicit strong cellular and humoral immune responses. However, the immune system response may vary according to the different factors, mainly because of mechanism of pathogencity (18). Therefore, the MEV was subjected to host immune simulation response analysis (79). Our candidate vaccine demonstrated the highest development of IFN-γ during immune simulation validation, with substantial IL-10 and IL-2 activities. There have also been noticed excess active immunoglobulins (i.e. IgG, IgM, and their isotypes which can be involved in the isotype switching). For efficient transporation into the host, vaccine candiate should posses potential binding capabalities with the host immune system receptors, such as TLR2 and TLR3 (80). Molecular Docking and 100 ns MD simulation not only verified the strong interactions between TLRs and MEV but also showed that a very small amount of energy was required for this stable binding in the MMGB(PB)SA analysis. During MD simulations, minor fluctuations were observed. Thus, these results suggested that the MEV will be able to strongly bind with immune receptors and effectively transported into the body. Vaccines developed through conventional techniques are more effective if subject to the immune system of model organisms but are found to be ineffective when administered to humans due to the complex nature of the human immune system (81). Therefore, using reliable reverse vaccinology, vaccine informatics and biophysics approaches, a safe, specific, and highly effective vaccine candidate was developed in this scientific study that could provide long-term immunity against CCHFV.

## Conclusions

In the current study, we presented an integrated computational framework describing the design of an MEV by targeting major antigenic/conserved proteins of the CCHFV. The formulated MEV strongly elicit both primary and secondary immune responses, showed a good affinity of binding with innate immunity TLR2 and TLR3 receptors, thus providing the adaptive immunity to establish and counter the pathogen properly. The intermolecular affinity was validated by molecular dynamics simulations that predicted highly stable binding of the MEV with the receptors, which was evident by strong chemical interactions. Despite keeping high standards in the computational methodology for MEV design against CCHFV, there are still several limitations that can be improved and investigated in future studies. For example, the vaccine is immunogenic but the real extent of immune protection against the pathogen is something that needs to be explored experimentally. The current results are preliminary and still need to be uncovered *in vitro* and *in vivo*; however, the selection criteria for filtering epitopes and post analysis were quite stringent, which facilitate the designed MEV to be the potent candidate against CCHFV. In a nutshell, the presented MEV construct must be evaluated experimentally to uncover its real immunogenicity for practical applications.

## Data Availability Statement

The original contributions presented in the study are included in the article/**Supplementary Material**. Further inquiries can be directed to the corresponding authors.

## Author Contributions

MQ, SI, and SA: conceptualization, data curation, visualization, software, methodology, investigation, and writing—original draft preparation. MM, SA, and UA: validation and writing—reviewing and editing. L-LC: supervision, project administration, funding, and writing—reviewing and editing. All authors contributed to the article and approved the submitted version.

## Funding

This work was supported by the starting research grant for High-level Talents from Guangxi University, China, and Postdoctoral Project from Guangxi University, China.

## Conflict of Interest

The authors declare that the research was conducted in the absence of any commercial or financial relationships that could be construed as a potential conflict of interest.
