# Supplementary material for: Development of a Novel Multi-Epitope Vaccine Against Crimean-Congo Hemorrhagic Fever Virus: An Integrated Reverse Vaccinology, Vaccine Informatics and Biophysics Approach
Source: Front Immunol. 2021 Jun 16;12:669812. doi: 10.3389/fimmu.2021.669812 (PMC8242340; doi:10.3389/fimmu.2021.669812)
Supplement: Supplementary file 1 [file DataSheet_1.docx]

**Figure S1.** Population coverage by the final set of epitopes.


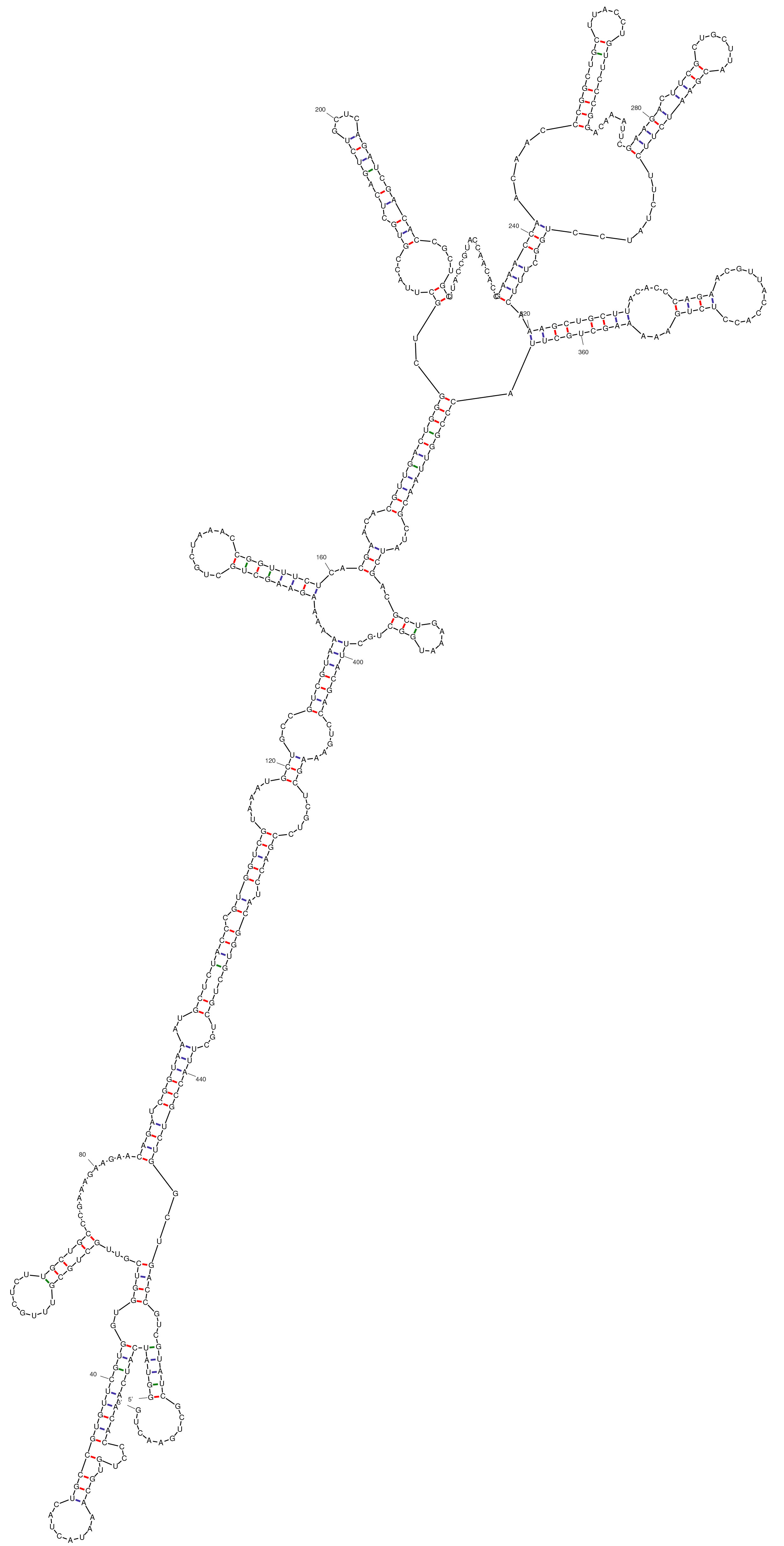


**Figure S2.** Secondary structure of MEV optimized sequence mRNA

**Table S1.** MEV refined models prioritized based on the lowest galaxy energy. Structural statistics of the initial unrefined model is also given.

| **Model** | **RMSD** | **MolProbity** | **Clash score** | **Poor rotamers** | **Rama favored** | **GALAXY energy** |
| --- | --- | --- | --- | --- | --- | --- |
| Initial | 0 | 3.522 | 145.6 | 2.4 | 87.7 | 12203.27 |
| MODEL 1 | 4.214 | 1.266 | 1.9 | 0 | 95.5 | -3151.35 |
| MODEL 2 | 4.372 | 1.523 | 3.4 | 0 | 94.2 | -3105.52 |
| MODEL 3 | 0.99 | 1.248 | 1.5 | 0 | 94.8 | -3096.46 |
| MODEL 4 | 3.931 | 1.356 | 3 | 0 | 96.1 | -3090.16 |
| MODEL 5 | 1.007 | 1.029 | 0.4 | 0 | 94.2 | -3080.71 |
| MODEL 6 | 3.478 | 1.448 | 3 | 0 | 94.8 | -3079.82 |
| MODEL 7 | 3.911 | 1.475 | 2.6 | 0 | 93.5 | -3071.57 |
| MODEL 8 | 3.556 | 1.361 | 2.3 | 0 | 94.8 | -3067.26 |
| MODEL 9 | 4.264 | 1.522 | 3.8 | 0 | 94.8 | -3062.72 |
| MODEL 10 | 3.945 | 1.407 | 2.6 | 0 | 94.8 | -3060.87 |

**Table S2.** TLR2 refined PatchDock complexes.

| **Rank** | **Solution Number** | **Global Energy** | **Attractive VdW** | **Repulsive VdW** | **ACE** | **HB** |
| --- | --- | --- | --- | --- | --- | --- |
|  |  | ↓ |  |  |  |  |
| 1 | 5 | -14.33 | -42.75 | 32.11 | 7.58 | -5.46 |
| 2 | 8 | -4.04 | -3.91 | 0.82 | 2.10 | -2.24 |
| 3 | 3 | 2.66 | -24.16 | 11.24 | 10.72 | -1.19 |
| 4 | 7 | 12.10 | -13.47 | 5.18 | 3.53 | -1.93 |
| 5 | 2 | 29.00 | -1.54 | 0.00 | 2.89 | 0.00 |
| 6 | 1 | 38.81 | -9.70 | 3.68 | 8.76 | -1.25 |
| 7 | 9 | 67.52 | -30.98 | 36.81 | 15.76 | -3.34 |
| 8 | 6 | 70.90 | -15.52 | 92.10 | 7.00 | -1.08 |
| 9 | 4 | 195.27 | -18.64 | 213.18 | 15.43 | -0.79 |
| 10 | 10 | 3930.96 | -41.19 | 4906.37 | 5.16 | -6.42 |

*VdW (van der Waals), ACE (Atomic Contact Energy), HB (Hydrogen Bonding).

**Table S3.** TLR3 refined PatchDock complexes.

| **Rank** | **Solution Number** | **Global Energy** | **Attractive VdW** | **Repulsive VdW** | **ACE** | **HB** |
| --- | --- | --- | --- | --- | --- | --- |
|  |  | ↓ |  |  |  |  |
| 1 | 9 | -6.95 | -7.70 | 1.50 | -2.47 | 0.00 |
| 2 | 7 | -4.70 | -26.88 | 4.98 | 10.51 | -2.81 |
| 3 | 4 | -3.59 | -38.77 | 16.29 | 14.37 | -1.89 |
| 4 | 8 | -3.13 | -8.60 | 4.06 | 2.02 | -2.74 |
| 5 | 3 | 5.60 | -2.68 | 0.79 | 2.12 | 0.00 |
| 6 | 6 | 13.68 | -26.63 | 28.19 | 13.63 | -4.19 |
| 7 | 2 | 22.90 | -13.65 | 18.59 | 9.08 | -2.32 |
| 8 | 10 | 29.81 | -23.23 | 12.66 | 2.17 | -1.56 |
| 9 | 5 | 34.06 | -5.68 | 9.45 | 6.89 | -0.35 |
| 10 | 1 | 43.55 | -13.42 | 1.99 | 9.81 | -3.96 |

*VdW (van der Waals), ACE (Atomic Contact Energy), HB (Hydrogen Bonding).

**Table S4**. List of hotspot residues in MMGBSA and MMPBSA

| **MEV-TLR2** | | | | | | | | | | | |
| --- | --- | --- | --- | --- | --- | --- | --- | --- | --- | --- | --- |
| **MMGBSA** | | | | | | **MMPBSA** | | | | | |
| Total Energy | | Sidechain Energy | | Backbone Energy | | Total Energy | | Sidechain Energy | | Backbone Energy | |
| Ser33 | -5.4 | Ser33 | -4.4 | Ser33 | -1.0 | Ser33 | -6.7 | Ser33 | -5.7 | Ser33 | -1.0 |
| Ser56 | -6.7 | Ser56 | -5.7 | Ser56 | -1.0 | Ser56 | -7.1 | Ser56 | -7.0 | Ser56 | -0.1 |
| Lys55 | -2.7 | Lys55 | -2.0 | Lys55 | -0.7 | Lys55 | -4.8 | Lys55 | -3.8 | Lys55 | -1.0 |
| Gln79 | -7.1 | Gln79 | -5.8 | Gln79 | -1.3 | Gln79 | -3.8 | Gln79 | -3.5 | Gln79 | -0.3 |
| Gln152 | -3.4 | Gln152 | -2.9 | Gln152 | -0.5 | Gln152 | -1.9 | Gln152 | -1.5 | Gln152 | -0.4 |
| Asn177 | -8.4 | Asn177 | -7.4 | Asn177 | -1.0 | Asn177 | -7.5 | Asn177 | -7.0 | Asn177 | -0.5 |
| Val80 | -2.4 | Val80 | -2.2 | Val80 | -0.2 | Val80 | -8.4 | Val80 | -7.4 | Val80 | -1.0 |
| Gly344 | -9.4 | Gly344 | -8.7 | Gly344 | -0.7 | Gly344 | -5.9 | Gly344 | -3.9 | Gly344 | -2.0 |
| Asn345 | -1.4 | Asn345 | -1.2 | Asn345 | -0.2 | Asn345 | -4.8 | Asn345 | -3.8 | Asn345 | -1.0 |
| Arg422 | -1.9 | Arg422 | -1.7 | Arg422 | -0.7 | Arg422 | -3.8 | Arg422 | -3.2 | Arg422 | -0.6 |
| Tyr483 | -4.7 | Tyr483 | -4.0 | Tyr483 | -0.7 | Tyr483 | -6.4 | Tyr483 | -5.4 | Tyr483 | -1.0 |
| Val503 | -4.1 | Val503 | -3.1 | Val503 | -1.1 | Val503 | -5.7 | Val503 | -5.0 | Val503 | -0.7 |
| Lys505 | -4.8 | Lys505 | -3.7 | Lys505 | -1.1 | Arg422 | -5.9 | Arg422 | -4.9 | Arg422 | -1.0 |
| Arg507 | -6.1 | Arg507 | -6.0 | Arg507 | -6.0 |  |  |  |  |  |  |
| Val556 | -5.7 | Val556 | -5.1 | Val556 | -0.6 |  |  |  |  |  |  |
| Asp557 | -2.7 | Asp557 | -2.0 | Asp557 | -0.7 |  |  |  |  |  |  |
| Glu629 | -1.8 | Glu629 | -1.3 | Glu629 | -0.5 |  |  |  |  |  |  |
| **MEV-TLR3** | | | | | | | | | | | |
| **MMGBSA** | | | | | | **MMPBSA** | | | | | |
| Total Energy | | Sidechain Energy | | Backbone Energy | | Total Energy | | Sidechain Energy | | Backbone Energy | |
| Hie3 | -2.85 | Val1 | -1.84 | Hie3 | -1.61 | Arg454 | -8.09 | Val1 | -1.79 | Hie3 | -1.43 |
| Val5 | -1.15 | Hie3 | -1.24 | Thr137 | 0.00 | Arg453 | -7.83 | Asp7 | -1.88 | Leu175 | 0.00 |
| Asp7 | -2.49 | Val5 | -1.14 | Cys187 | 0.00 | Arg704 | -6.13 | Arg453 | -7.35 | Glu628 | -1.61 |
| Leu135 | 0.00 | Asp7 | -2.25 | Hie189 | 0.00 | Arg646 | -6.05 | Arg454 | -7.80 | Thr629 | -2.26 |
| Asn143 | 0.00 | Lys60 | 0.00 | Asn218 | 0.00 | Glu722 | -5.65 | Phe609 | -1.04 |  |  |
| Ala190 | 0.00 | Val115 | 0.00 | Trp244 | 0.00 | Lys637 | -4.09 | Phe622 | -2.73 |  |  |
| Ser207 | 0.00 | Val139 | 0.00 | Thr248 | 0.00 | Lys679 | -3.57 | Asn627 | -1.05 |  |  |
| Asn236 | 0.00 | Ser159 | 0.00 | Ser315 | 0.00 | Thr629 | -3.21 | Lys637 | -3.74 |  |  |
| Thr237 | 0.00 | Ser207 | 0.00 | Ser368 | 0.00 | Tyr638 | -3.14 | Tyr638 | -2.37 |  |  |
| Leu242 | 0.00 | Leu247 | 0.00 | Asp390 | 0.00 | Lys720 | -3.01 | Val642 | -1.31 |  |  |
| Met249 | 0.00 | Trp267 | 0.00 | Ala514 | 0.00 | Phe622 | -2.78 | Arg646 | -6.44 |  |  |
| Ala266 | 0.00 | Ser286 | 0.00 | Glu628 | -1.01 | Asp7 | -2.26 | Val649 | -1.19 |  |  |
| Gly291 | 0.00 | Cys321 | 0.00 | Cys686 | -1.44 | Hie3 | -2.08 | Lys679 | -3.38 |  |  |
| Ser337 | 0.00 | Leu343 | 0.00 |  |  | Val743 | -1.71 | Trp688 | -1.35 |  |  |
| Leu343 | 0.00 | Ser372 | 0.00 |  |  | Phe721 | -1.52 | Arg704 | -5.87 |  |  |
| Asp390 | 0.00 | Leu374 | 0.00 |  |  | Val642 | -1.45 | Lys720 | -3.29 |  |  |
| Arg453 | -5.83 | Gly414 | 0.00 |  |  | Trp688 | -1.39 | Phe721 | -1.67 |  |  |
| Arg454 | -5.91 | Gly419 | 0.00 |  |  | Val649 | -1.39 | Glu722 | -5.53 |  |  |
| Ser463 | 0.00 | Ala442 | 0.00 |  |  | Thr744 | -1.33 | Val743 | -1.37 |  |  |
| Thr603 | -2.01 | Arg453 | -5.71 |  |  | Hie683 | -1.23 | Thr744 | -1.13 |  |  |
| Phe609 | -1.15 | Arg454 | -6.04 |  |  | Cys686 | -1.11 |  |  |  |  |
| Ile619 | -1.06 | Pro543 | 0.00 |  |  | Gln694 | -1.11 |  |  |  |  |
| Phe622 | -3.47 | Thr603 | -2.13 |  |  | Arg692 | -1.10 |  |  |  |  |
| Asn627 | -1.04 | Phe609 | -1.21 |  |  | Val5 | -1.07 |  |  |  |  |
| Glu628 | -1.03 | Ile619 | -1.25 |  |  | Phe609 | -1.06 |  |  |  |  |
| Thr629 | -1.31 | Phe622 | -3.57 |  |  | Gly136 | 0.00 |  |  |  |  |
| Thr634 | -2.18 | Asn627 | -1.33 |  |  | Ile387 | 0.00 |  |  |  |  |
| Lys637 | -2.06 | Thr634 | -1.89 |  |  |  |  |  |  |  |  |
| Tyr638 | -3.80 | Lys637 | -2.53 |  |  |  |  |  |  |  |  |
| Arg641 | -1.92 | Tyr638 | -3.35 |  |  |  |  |  |  |  |  |
| Val642 | -1.72 | Arg641 | -2.12 |  |  |  |  |  |  |  |  |
| Arg646 | -5.03 | Val642 | -1.55 |  |  |  |  |  |  |  |  |
| Val649 | -1.50 | Arg646 | -5.63 |  |  |  |  |  |  |  |  |
| Lys679 | -2.72 | Val649 | -1.38 |  |  |  |  |  |  |  |  |
| Hie683 | -1.40 | Lys679 | -2.83 |  |  |  |  |  |  |  |  |
| Cys686 | -2.32 | Hie683 | -1.35 |  |  |  |  |  |  |  |  |
| Trp688 | -2.95 | Trp688 | -2.96 |  |  |  |  |  |  |  |  |
| Gln694 | -1.67 | Gln694 | -1.53 |  |  |  |  |  |  |  |  |
| Arg704 | -5.84 | Arg704 | -6.02 |  |  |  |  |  |  |  |  |
| Lys720 | -1.06 | Lys720 | -2.36 |  |  |  |  |  |  |  |  |
| Phe721 | -1.24 | Phe721 | -1.37 |  |  |  |  |  |  |  |  |
| Glu722 | -6.29 | Glu722 | -6.22 |  |  |  |  |  |  |  |  |
| Val743 | -2.01 | Val743 | -1.62 |  |  |  |  |  |  |  |  |
| Thr744 | -1.11 | Thr744 | -1.15 |  |  |  |  |  |  |  |  |
